# Supplementary material for: Restoring Axonal Organelle Motility and Regeneration in Cultured FUS-ALS Motoneurons through Magnetic Field Stimulation Suggests an Alternative Therapeutic Approach
Source: Cells. 2023 May 29;12(11):1502. doi: 10.3390/cells12111502 (PMC10252208; doi:10.3390/cells12111502)
Supplement: Supplementary file 1 [file cells-12-01502-s001.zip › cells-2303008-supplementary.pdf]

Supplements

Contents:

- Supplemental Tables S1 and S2
- Supplemental Figures S1-S10
- Movie S1 and S2

**Mean speed of mitochondria**	**Mean rank Diff.**	**Z**	**P-value**	**Summary**
distal untreated Ctrl vs distal untreated mutant FUS	8797.02726	19.17492	7.20E-80	****
distal untreated mutant FUS vs distal 2 Hz mutant FUS	-440.50321	-0.95909	1	ns
distal untreated mutant FUS vs distal 10 Hz mutant FUS	-3339.0986	-6.94724	4.47E-10	****
distal untreated mutant FUS vs distal 10/2 Hz mutant FUS	-10353.9792	-24.4062	1.77E-129	****
distal untreated mutant FUS vs proximal untreated mutant FUS	-3748.37658	-8.60451	9.20E-16	****
proximal untreated Ctrl vs proximal untreated mutant FUS	5982.34582	17.6184	2.14E-67	****
proximal untreated mutant FUS vs proximal 2 Hz mutant FUS	1745.12635	4.95283	8.78E-05	****
proximal untreated mutant FUS vs proximal 10 Hz mutant FUS	-7600.57211	-24.1424	1.08E-126	****
proximal untreated mutant FUS vs proximal 10/2Hz mutant FUS	-997.82891	-2.68874	0.86067	ns
**% Moving tracks of mitochondria**	**Mean rank Diff.**	**Z**	**P-value**	**Summary**
distal untreated Ctrl vs distal untreated mutant FUS	118.0897	4.37076	0.00149	**
distal untreated mutant FUS vs distal 2 Hz mutant FUS	-104.968	-4.07968	0.00541	**
distal untreated mutant FUS vs distal 10 Hz mutant FUS	-63.6259	-2.47288	1	ns
distal untreated mutant FUS vs distal 10/2 Hz mutant FUS	-99.5753	-4.19139	0.00333	**
distal untreated mutant FUS vs proximal untreated mutant FUS	-154.61371	-5.36891	9.51E-06	****
proximal untreated Ctrl vs proximal untreated mutant FUS	16,65611	0,54471	1	ns
proximal untreated mutant FUS vs proximal 2 Hz mutant FUS	28,84389	0,94329	1	ns
proximal untreated mutant FUS vs proximal 10 Hz mutant FUS	15,32265	0,50724	1	ns
proximal untreated mutant FUS vs proximal 10/2Hz mutant FUS	-6,24434	-0,20421	1	ns
**Mean speed of lysosomes**	**Mean rank Diff.**	**Z**	**P-value**	**Summary**

distal untreated Ctrl vs distal untreated mutant FUS	1800.56914	4.47904	9.00E-04	***
distal untreated mutant FUS vs distal 2 Hz mutant FUS	-1201.83336	-4.00683	0.00738	**
distal untreated mutant FUS vs distal 10 Hz mutant FUS	-1591.0924	-5.17863	2.68E-05	****
distal untreated mutant FUS vs distal 10/2 Hz mutant FUS	-5465.13092	-19.3476	2.56E-81	****
distal untreated mutant FUS vs proximal untreated mutant FUS	-2507.02931	-9.24475	2.83E-18	****
proximal untreated Ctrl vs proximal untreated mutant FUS	1090.89834	2.73625	0.74572	ns
proximal untreated mutant FUS vs proximal 2 Hz mutant FUS	-1030,09	-4,98511	7,43E-05	****
proximal untreated mutant FUS vs proximal 10 Hz mutant FUS	-5082,3	-27,8562	1,09E-168	****
proximal untreated mutant FUS vs proximal 10/2Hz mutant FUS	-2214,19	-10,6654	1,77E-24	****
**% Moving tracks of lysosomes**	**Mean rank Diff.**	**Z**	**P-value**	**Summary**
distal untreated Ctrl vs distal untreated mutant FUS	85.12908	3.12905	0.21045	ns
distal untreated mutant FUS vs distal 2 Hz mutant FUS	-93.9005	-3.62432	0.03477	*
distal untreated mutant FUS vs distal 10 Hz mutant FUS	-65.3478	-2.52226	1	ns
distal untreated mutant FUS vs distal 10/2 Hz mutant FUS	-61.2324	-2.55963	1	0
distal untreated mutant FUS vs proximal untreated mutant FUS	-139.548	-5.03135	5.84E-05	****
proximal untreated Ctrl vs proximal untreated mutant FUS	-34,5824	-1,16813	1	ns
proximal untreated mutant FUS vs proximal 2 Hz mutant FUS	-30,6824	-1,0364	1	ns
proximal untreated mutant FUS vs proximal 10 Hz mutant FUS	-26,8556	-0,91918	1	ns
proximal untreated mutant FUS vs proximal 10/2Hz mutant FUS	2,58235	0,08723	1	ns

Table S1. Statistical analysis of mean speed of mitochondria and lysosomes as well as percent moving tracks of mitochondria and lysosomes. Corresponds to figure 2c–f. One-way ANOVA with Kruskal-Wallis post-hoc test was utilized for revealing significance differences in pairwise comparisons of the different MS treatment conditions (untreated, 2 Hz only, 10 Hz only, 10/2 Hz). Asterisks: highly significant alteration in indicated pairwise comparison, * $p \leq 0.05$, **$p \leq 0.01$, ***$p \leq 0.001$, ****$p \leq 0.0001$, ns: no significant difference.

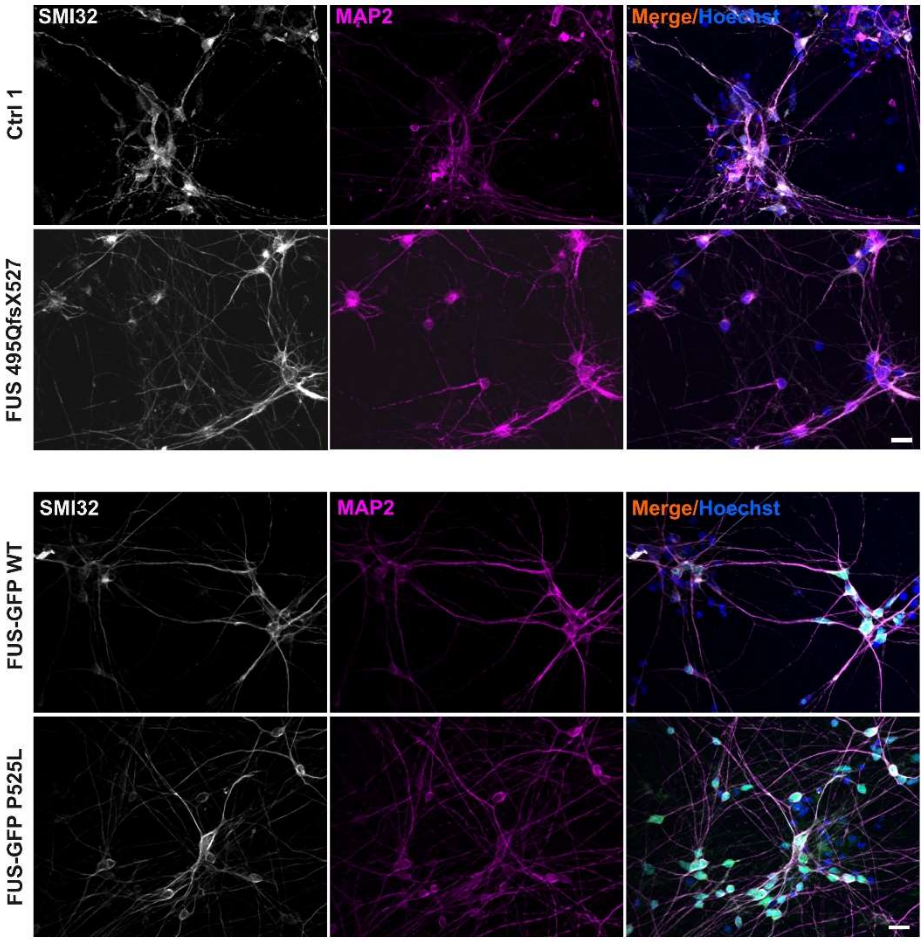

Figure S1. Motoneuronal characterization by IF microscopy of selected individual untreated Ctrl and mutant FUS-ALS cell lines used for the pooled analysis in corresponding Fig. 1b–e. Scale bar = 20µm.

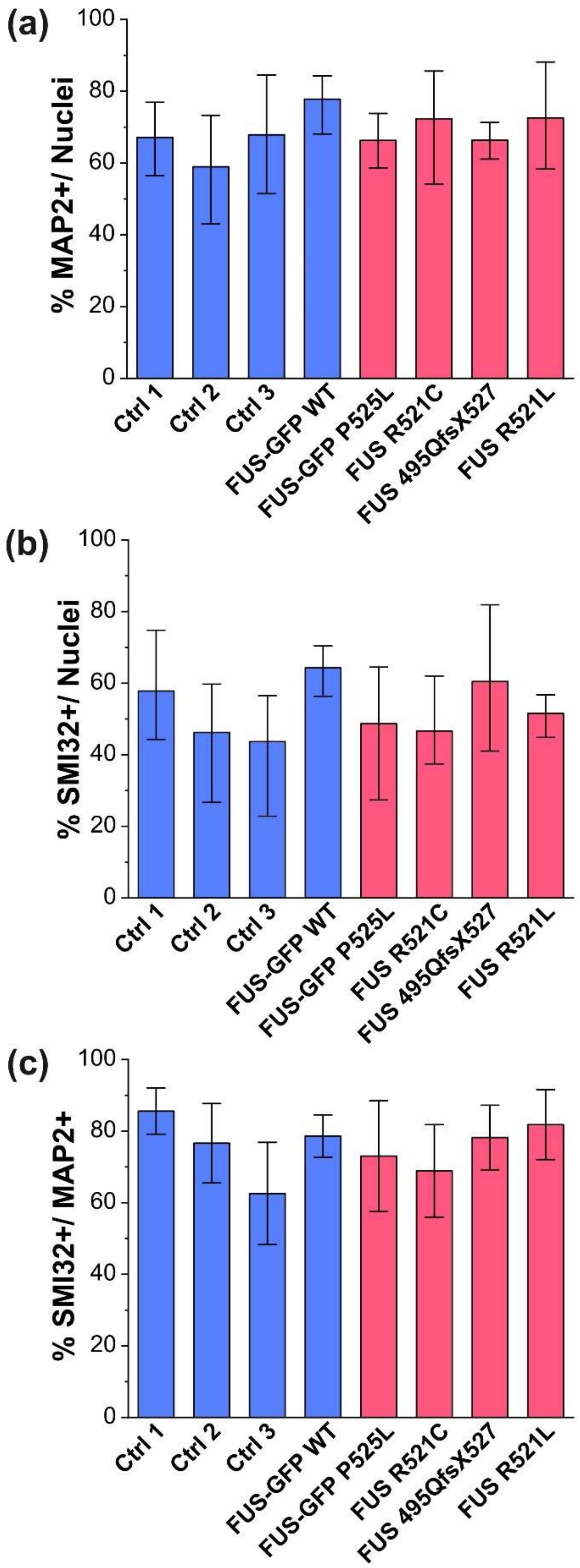

Figure S2. Quantitative image analysis of individual untreated Ctrl and mutant FUS-ALS MN cell lines corresponding to the pooled motoneuronal analysis in Fig. 1c–e. Means ± SD. One-way ANOVA with Bonferroni post-hoc test revealed no significant alteration in any pairwise comparison.

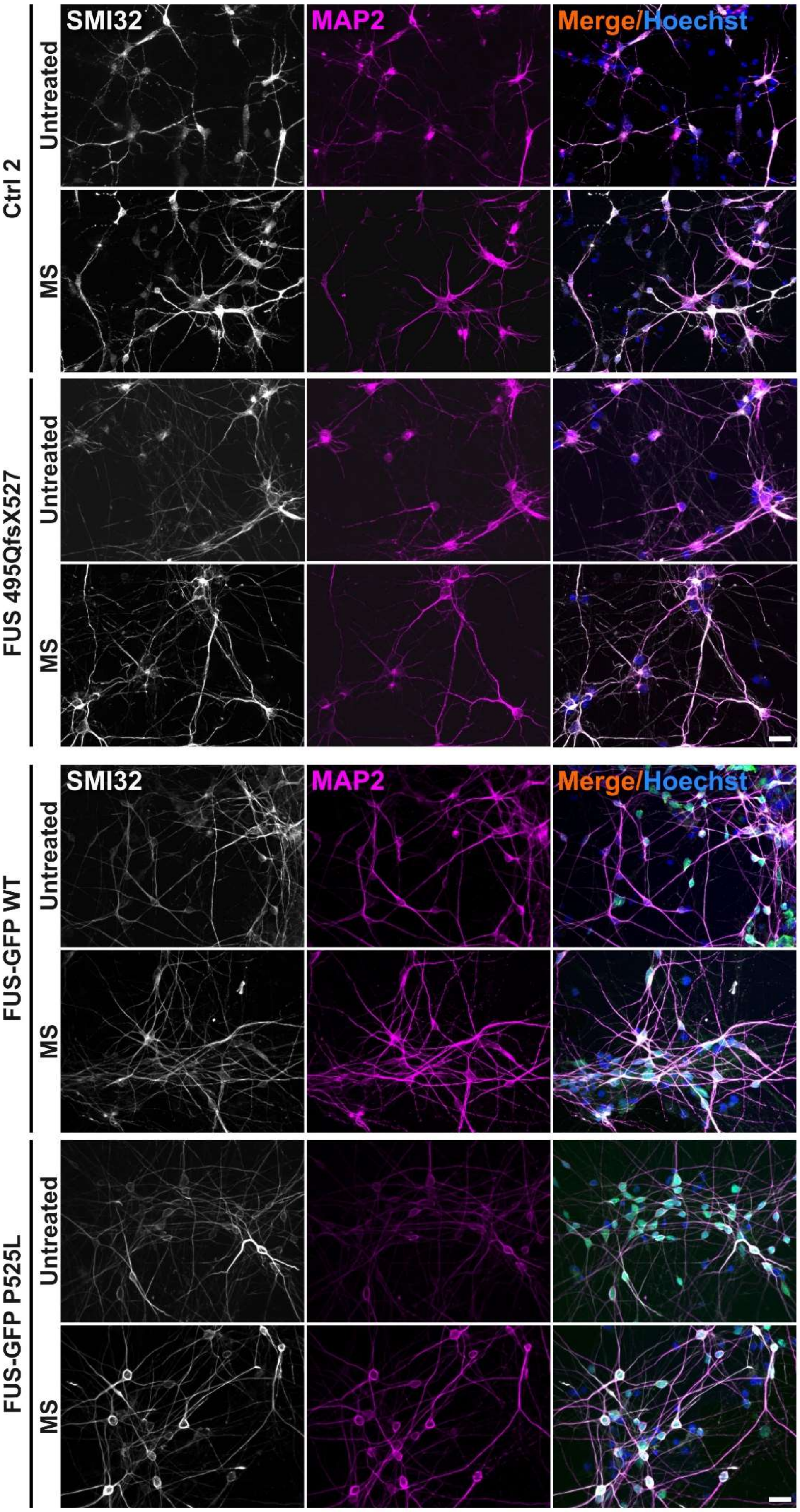
SMI32
MAP2
Merge/Hoechst
Ctrl 2
Untreated
MS
FUS 495QfsX527
Untreated
MS
SMI32
MAP2
Merge/Hoechst
FUS-GFP WT
Untreated
MS
FUS-GFP P525L
Untreated
MS

Figure S3 Motoneuronal characterization by IF microscopy of individual Ctrl and mutant FUS-ALS MN cell lines either untreated or after MS at 10 Hz used for the pooled analysis in corresponding Fig. 1f–h. Scale bar = 20μm.

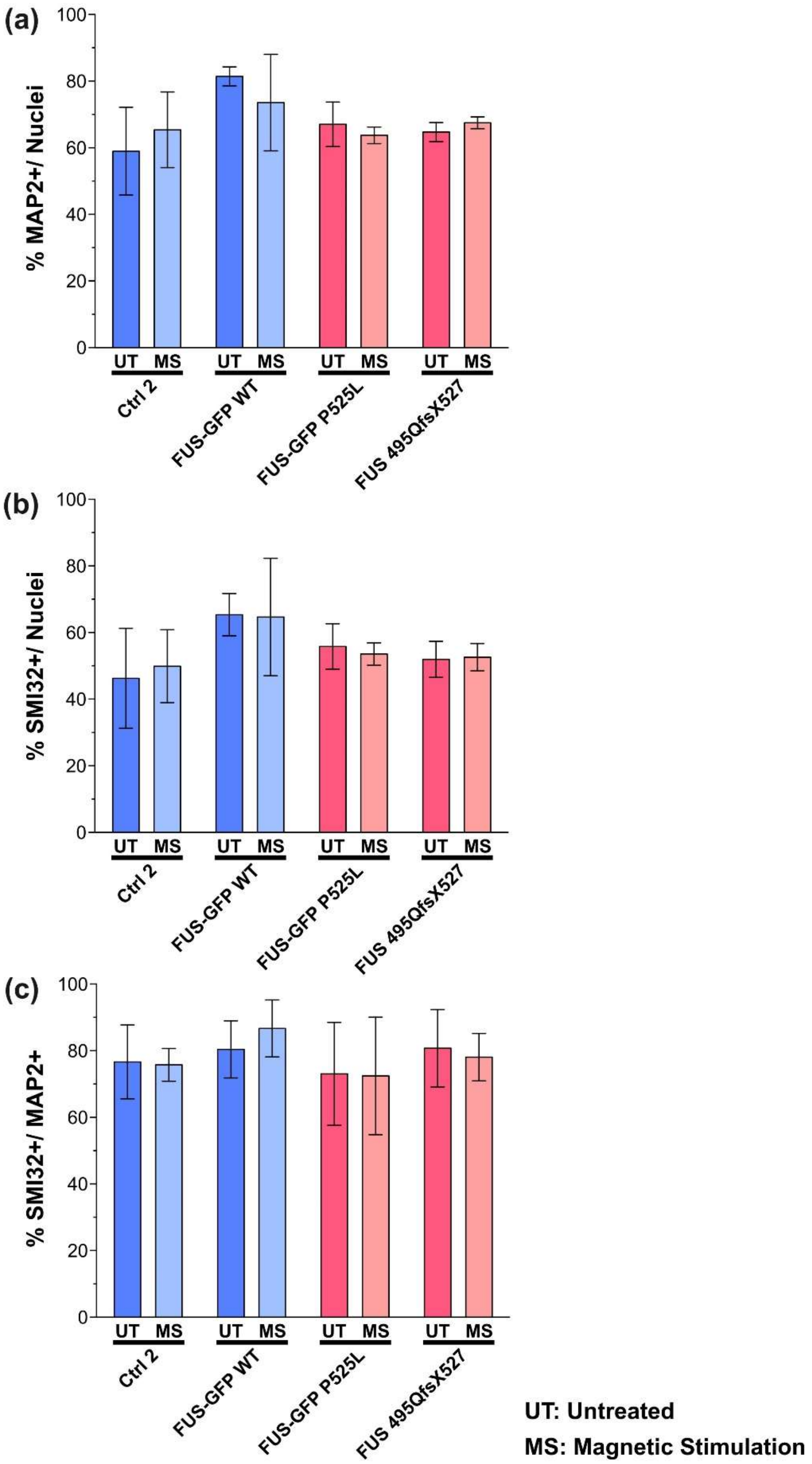

Figure S4. Quantitative image analysis of the effect of MS on motoneuronal maturation on individual Ctrl and mutant FUS-ALS cell lines corresponding to the pooled analysis in Fig. 1f–h. One-way ANOVA with Bonferroni post-hoc test revealed no significant alteration in any pairwise comparison.

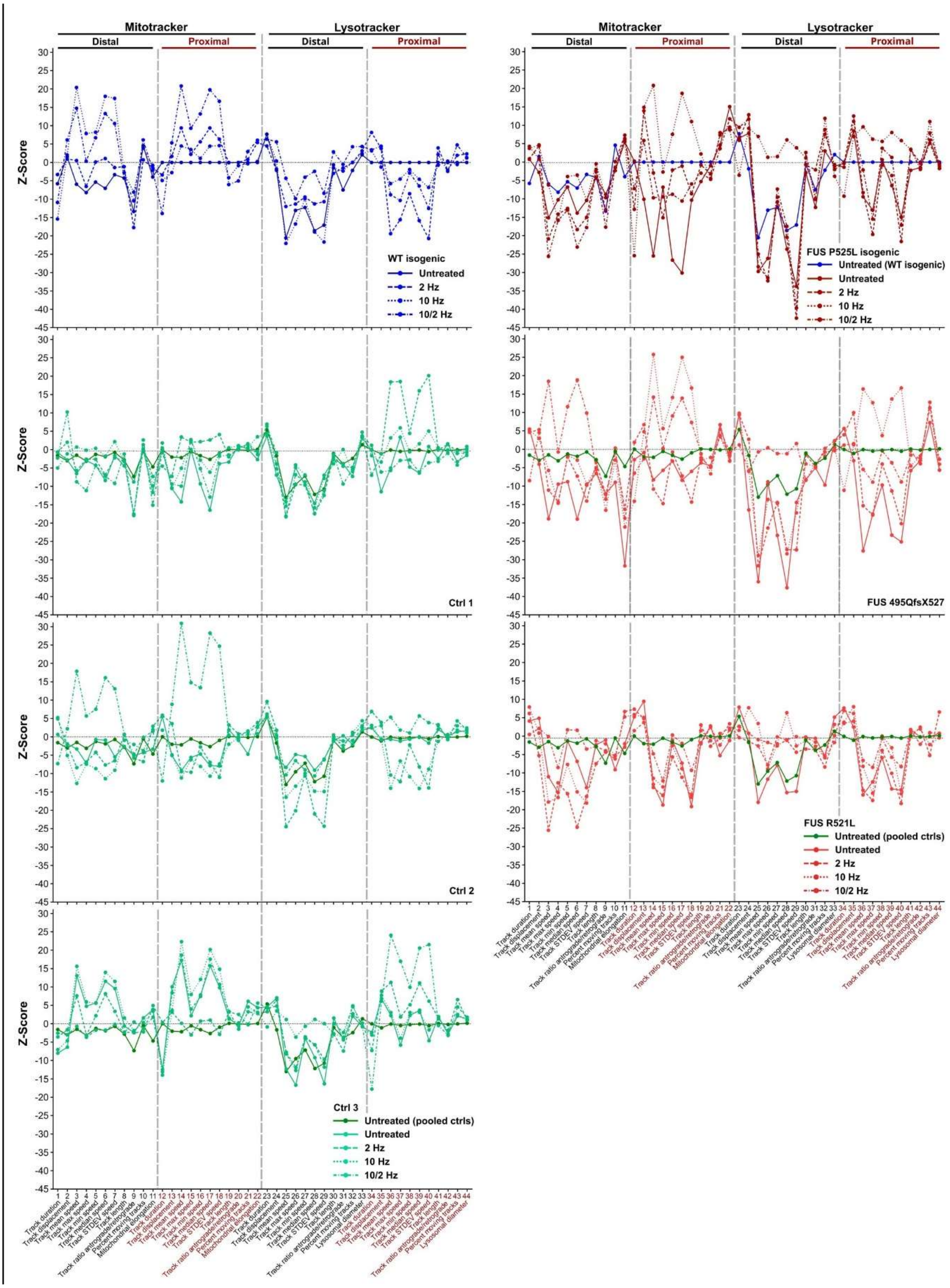

Figure S5. Multiparametric HC profiles of individual Ctrl and mutant FUS-ALS cell lines that were pooled for the corresponding profiles in Fig. 3a–c. Axonal trafficking in response to MS with different frequencies are presented in dashed lines (2 Hz), dotted lines (10 Hz) as well as dashed and dotted lines (combinatorial sequence of 10 Hz and 2 Hz, 10/2 Hz) versus untreated cells (straight lines).

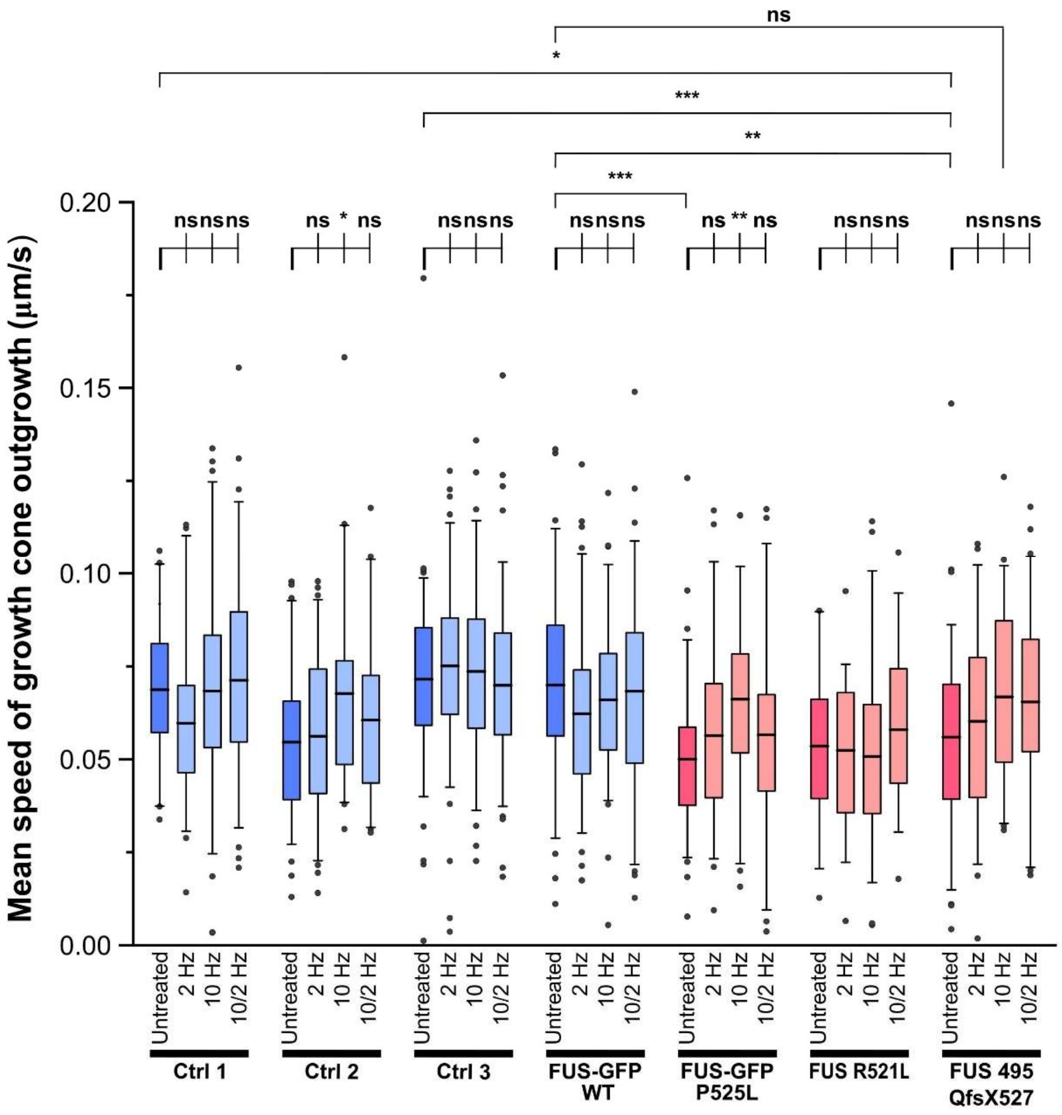

Figure S6. Mean speed of growth cone outgrowth in individual Ctrl and mutant FUS-ALS cell lines that were pooled for the corresponding box plots in Fig. 4c. Center lines are means, whiskers span 95% of data points of per-growth-cone values. One-way ANOVA with Bonferroni post-hoc test was utilized for revealing significance differences in pairwise comparisons of the different MS treatment conditions (untreated, 2 Hz only, 10 Hz only, 10/2 Hz). Asterisks: highly significant alteration in indicated pairwise comparison, * $p \leq 0.05$, **$p \leq 0.01$, ***$p \leq 0.001$, ns: no significant difference.

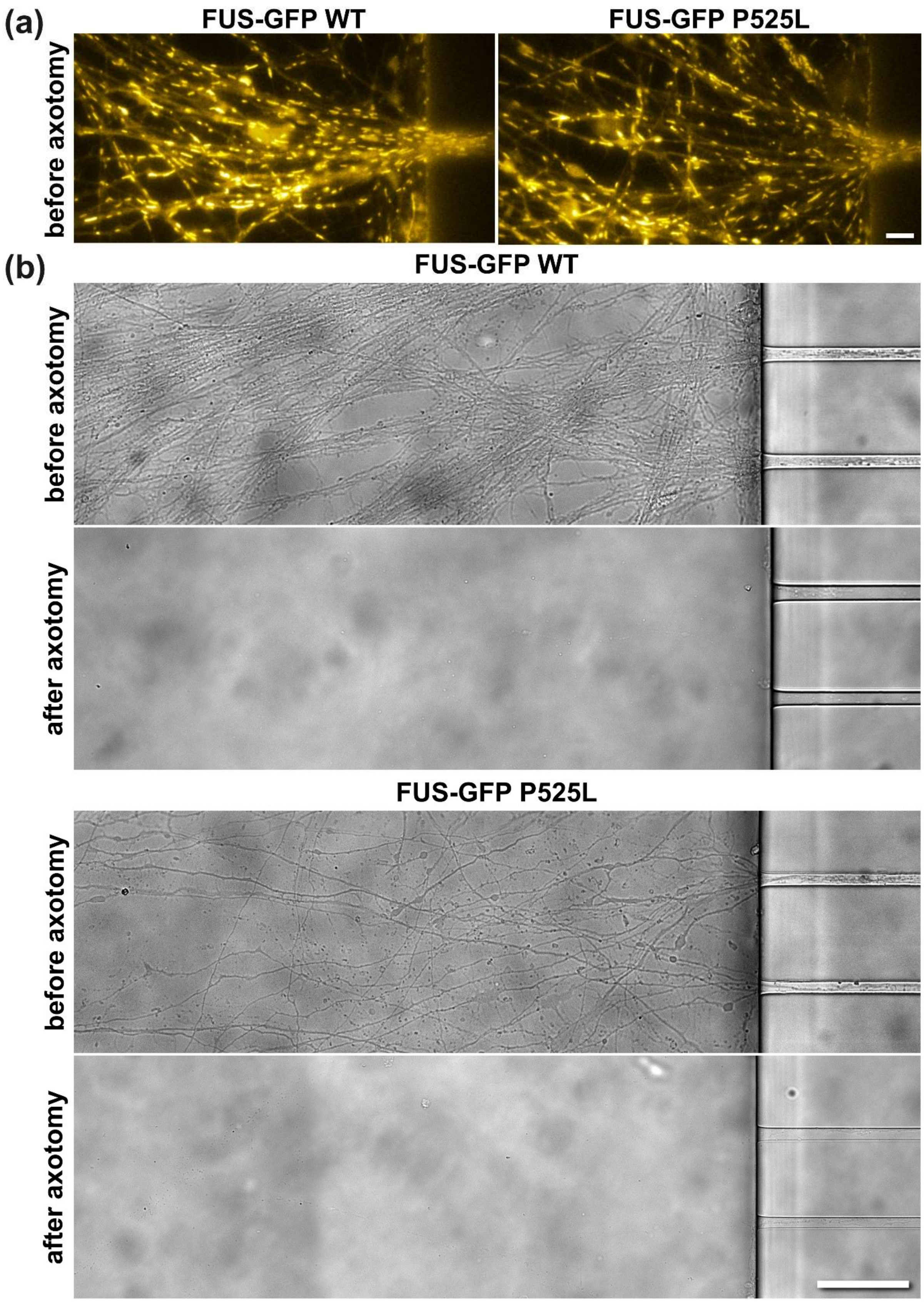

Figure S7. Documentation of complete axotomy. Shown as representative examples are the FUS-GFP WT versus FUS-GFP P525L MN lines (Table 1). (a) Staining with Mitotracker Deep

Red revealed how the axons were sprouting out from the distal microchannel exits in compartmentalized MNs cultures before the axotomy was performed. Wide field fluorescent live imaging was performed with an oil immersion objective at 100x magnification (NA 1.4). Scale bar = 10 μm. (b) Brightfield images at 20x magnification from the same lines as in panel a immediately before and after the axotomy was performed. After successful axotomy, all axons and debris were completely removed, thereby enabling specific imaging of the subsequent axon regeneration, i.e. new axon outgrowth to the blank assay area as shown in Fig. 4b. Scale bar = 50 μm.

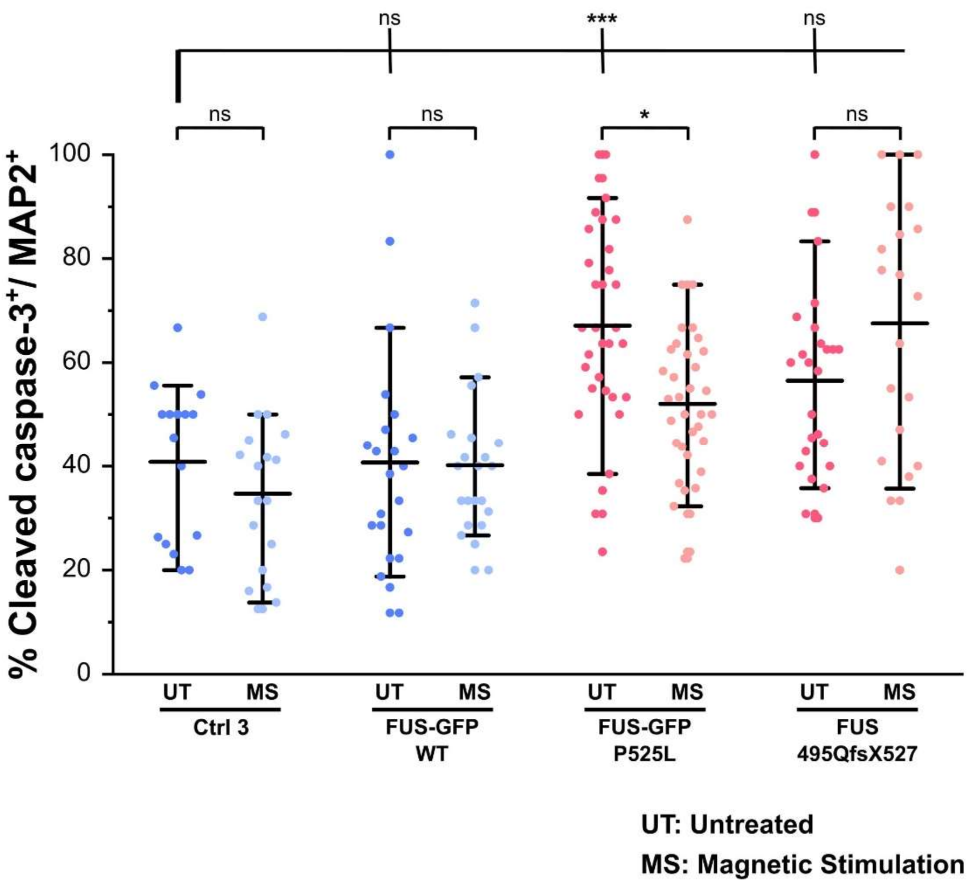

Figure S8. The effect of MS on neuronal survival in individual Ctrl and mutant FUS-ALS MN cell lines that were pooled for the corresponding immunofluorescence image analysis in Fig. 5c. Means ± SD. Percentages of cleaved caspase-3 IF intensities after MS were quantified in MAP2-positive neurons only and are shown as scatter dot plots with per-image values. One-way ANOVA with post-hoc Bonferroni test was utilized for revealing significance differences in pairwise comparisons. Asterisks: highly significant alteration in indicated pairwise comparison, *p ≤ 0.05, ***p ≤ 0.001****, p ≤ 0.0001, ns: no significant difference.

**% Cleaved caspase-3^{+}/ MAP2^{+}, pooled lines**	**Mean diff.**	**Z**	**P-value**	**Summary**
Ctrl pool MS vs Ctrl pool UT	-2,54497	-0,59303	1	ns
FUS pool UT vs Ctrl pool UT	20,83169	5,30316	2,00E-06	****
FUS pool UT vs Ctrl pool MS	23,37666	6,04941	4,97E-08	****
FUS pool MS vs Ctrl pool UT	15,08197	3,76993	0,00133	***
FUS pool MS vs Ctrl pool MS	17,62694	4,47623	8,09E-05	****
FUS pool MS vs FUS pool UT	-5,74972	-1,62494	0,63561	ns
**% Cleaved caspase-3^{+}/ MAP2^{+}, individual lines**	**Mean diff.**	**Z**	**P-value**	**Summary**
Ctrl 3 MS vs Ctrl 3 UT	-6,11694	-0,98369	1	ns
FUS-GFP WT UT vs Ctrl 3 UT	-0,11915	-0,02004	1	ns
FUS-GFP WT UT vs Ctrl 3 MS	5,99779	1,04274	1	ns
FUS-GFP WT MS vs Ctrl 3 UT	-0,624	-0,10494	1	ns
FUS-GFP WT MS vs Ctrl 3 MS	5,49294	0,95497	1	ns
FUS-GFP WT MS vs FUS-GFP WT UT	-0,50485	-0,09252	1	ns

FUS-GFP P525L UT vs Ctrl 3 UT	26,28739	4,76796	1,06E-04	***
FUS-GFP P525L UT vs Ctrl 3 MS	32,40433	6,11051	1,62E-07	****
FUS-GFP P525L UT vs FUS-GFP WT UT	26,40654	5,30109	9,16E-06	****
FUS-GFP P525L UT vs FUS-GFP WT MS	26,91139	5,40243	5,64E-06	****
FUS-GFP P525L MS vs Ctrl 3 UT	11,27749	2,0739	1	ns
FUS-GFP P525L MS vs Ctrl 3 MS	17,39443	3,32941	0,02944	ns
FUS-GFP P525L MS vs FUS-GFP WT UT	11,39664	2,32698	0,58954	ns
FUS-GFP P525L MS vs FUS-GFP WT MS	11,90148	2,43006	0,44959	ns
FUS-GFP P525L MS vs FUS-GFP P525L UT	-15,0099	-3,44134	0,02006	*
FUS 495QfsX527 UT vs Ctrl 3 UT	15,73237	2,71518	0,20313	ns
FUS 495QfsX527 UT vs Ctrl 3 MS	21,84931	3,9055	0,00369	**
FUS 495QfsX527 UT vs FUS-GFP WT UT	15,85152	2,99619	0,0871	ns
FUS 495QfsX527 UT vs FUS-GFP WT MS	16,35637	3,09162	0,06441	ns
FUS 495QfsX527 UT vs FUS-GFP P525L UT	-10,555	-2,19957	0,81433	ns
FUS 495QfsX527 UT vs FUS-GFP P525L MS	4,45489	0,9455	1	ns
FUS 495QfsX527 MS vs Ctrl 3 UT	26,7525	4,40712	4,97E-04	***
FUS 495QfsX527 MS vs Ctrl 3 MS	32,86945	5,59008	2,26E-06	****
FUS 495QfsX527 MS vs FUS-GFP WT UT	26,87166	4,80577	8,93E-05	****
FUS 495QfsX527 MS vs FUS-GFP WT MS	27,3765	4,89605	5,97E-05	****
FUS 495QfsX527 MS vs FUS-GFP P525L UT	0,46512	0,09069	1	ns
FUS 495QfsX527 MS vs FUS-GFP P525L MS	15,47502	3,06598	0,0699	ns
FUS 495QfsX527 MS vs FUS 495QfsX527 UT	11,02013	2,0297	1	ns

Table S2. Statistical analysis of percentages of cleaved caspase-3-positive neurons within the MAP2-positive cell population. Corresponds to Fig. 5c and S8. One-way ANOVA with Bonferroni post-hoc test was utilized for revealing significance differences in pairwise comparisons of pooled and individual cell lines, untreated (UT) versus magnetically stimulated (MS). Asterisks: highly significant alteration in indicated pairwise comparison, * $p \leq 0.05$, **$p \leq 0.01$, ***$p \leq 0.001$, ****$p \leq 0.0001$, ns: no significant difference.

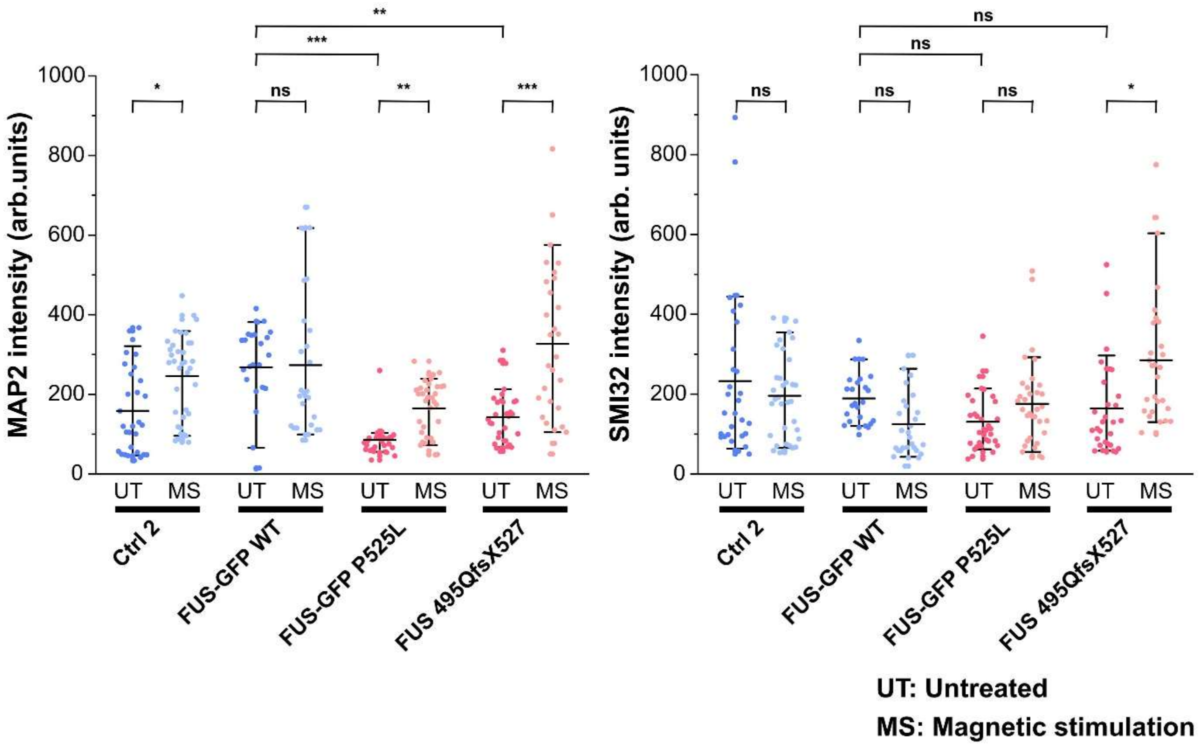

Figure S9. Amount of cytoskeletal markers normalized by total neurite length in response to MS in individual Ctrl and mutant FUS-ALS cell lines that were pooled for the corresponding immunofluorescence image analysis in Fig. 6c, d. Center lines are means, whiskers span 95% of data points of per-image values. One-way ANOVA with Kruskal-Wallis post-hoc test was utilized for revealing significance differences in pairwise comparisons. Asterisks: highly significant alteration in indicated pairwise comparison, * $p \leq 0.05$, **$p \leq 0.01$, ***$p \leq 0.001$ and ns: no significant difference.

FIJI macro "Growth cone characteristics"

```
setBatchMode(true);
starttime = getTime();
run("Bio-Formats Macro Extensions");
dir = getDirectory("Choose a Directory ");
run("Options...", "iterations=1 count=1 black");
run("Colors...", "foreground=white background=black selection=yellow"); //set colors
run("Set Measurements...", " center display redirect=None decimal=5");
if (roiManager("count")>0) {
	roiManager("Delete");
}
run("Clear Results");
files = newArray();
files = find(dir, files);
```

```
print(files.length);
for (i=0; i<files.length; i++) {
		open(files[i]);
		setBatchMode("show");
		ID=getImageID();
		waitForUser("Please select a growth cone by drawing a box around it. Make sure, the growth
cone stays inside the box for the whole video.");
		run("Duplicate...", "duplicate");
		id=getImageID();
		conetracking(files,i,ID,id);
		run("Close All");
}
endtime = getTime();
time = endtime-starttime;
waitForUser("We did it, team! In " + time/60000);

function find(dir, files) {
   list = getFileList(dir);
   for (i=0; i<list.length; i++) {
		if (endsWith(list[i], "/"))
      files = find(""+dir+list[i], files);
				else if (endsWith(list[i], ".tif")) {
						files = Array.concat(files,""+dir+list[i]); }
		}
		return files;
}

function conetracking(files,i,ID,id) {
		selectImage(id);
		getDimensions(width, height, channels, slices, frames);
		arrayname=newArray("frame","XM","YM");
		allresults=newArray("frame","XM","YM");
		run("Slice Keeper", "first=1 last=1 increment=1");
		rename("initial");
		run("Enhance Contrast...", "saturated=0.1 normalize");
		run("Subtract Background...", "rolling=20 light");
		run("Duplicate...", "title=[detect]");
		setAutoThreshold("Percentile dark no-reset");
		setOption("BlackBackground", true);
		run("Convert to Mask");
		run("Create Selection");
		run("Make Inverse");
		run("Set Measurements...", "Area display redirect=None decimal=5");
		run("Measure");
		size = getResult("Area",0);
		run("Clear Results");
		p = 0;
```

```
for (l=0; l<10; l++) {
	if (p==0) {
		selectWindow("detect");
		run("Duplicate...", "title=[detect2]");
		run("Convert to Mask");
		run("Create Selection");
		run("Make Inverse");
		enlarge_arg = "enlarge="+(-1+(l*0.1));
		run("Enlarge...", enlarge_arg);
		run("Measure");
		new_size = getResult("Area",0);
		run("Clear Results");
		enlarge_arg = "enlarge="+(1+(l*0.1));
		run("Enlarge...", enlarge_arg);
		if (new_size != size) {
							run("Set Measurements...", " center
			display redirect=None decimal=5");
			run("Clear Outside");
			run("Fill");
			run("Analyze Particles...", "clear add");
			selectWindow("initial");
			roiManager("show all with labels");
			setBatchMode("show");
			if (getBoolean("Is your desired growth cone detected?"))
{
				waitForUser("Please select the growthcone you want to
				track");
				roi = roiManager("index");
				p=1;
			}
		}
		selectWindow("detect2");
		close();
	}
}
selectWindow("detect");
roiManager("select", roi);
run("Fill");
run("Clear Outside");
roiManager("deselect");
roiManager("delete");
run("Analyze Particles...", "clear add");
roiManager("measure");
getPixelSize(unit, pw, ph, pd);
x=getResult("XM", 0)/pw;
y=getResult("YM", 0)/ph;
close();
```

```
selectWindow("initial");
close();
if (roiManager("count")>0) {
	roiManager("Delete");
}
run("Clear Results");
ytable = newArray();
xtable = newArray();
dist = newArray();
timepoint = newArray();
for (currframe = 0; currframe < frames; currframe++) {
	selectImage(id);
	Slice_arg = "first="+(currframe+1)+" last="+(currframe+1)+" increment=1";
	run("Slice Keeper", Slice_arg);
	run("Subtract Background...", "rolling=20 light");
	rename(currframe);
	setAutoThreshold("Percentile dark no-reset");
	setOption("BlackBackground", true);
	run("Convert to Mask");
	run("Create Selection");
	run("Make Inverse");
	run("Set Measurements...", "Area display redirect=None decimal=5");
	run("Measure");
	size = getResult("Area",0);
	run("Clear Results");
	p = 0;
			for (l=0; l<10; l++) {
				if (p==0) {
					enlarge_arg = "enlarge="+(-1+(l*0.1));
					run("Enlarge...", enlarge_arg);
					run("Measure");
					new_size = getResult("Area",0);
					run("Clear Results");
					if (new_size != size) {
						p=1;
					}
				}
			}
	run("Set Measurements...", " center display redirect=None decimal=5");
	run("Clear Outside");
	run("Fill");
	rank = expand(x,y,pw,ph);
	if (nResults!=0) {
					dist = Array.concat (dist, (sqrt(pow( (x*pw)-
		getResult("XM", rank),2)+pow( (y*ph)-getResult("YM", rank),2) ) ) );
		x=(getResult("XM", rank)/pw);
		y=(getResult("YM", rank)/ph);
```

```
                    ytable = Array.concat(ytable, getResult("YM", rank));
                    xtable = Array.concat(xtable, getResult("XM", rank));
            } else {
                    dist = Array.concat (dist, 0);
                    ytable = Array.concat(ytable, (y*ph));
                    xtable = Array.concat(xtable, (x*pw));
            }
            timepoint = Array.concat (timepoint, currframe);
            selectWindow(currframe);
            close();
            run("Clear Results");
        }
        for(t=0; t< xtable.length; t++) {
            setResult("Timepoint", t, timepoint[t]);
            setResult("X", t, xtable[t]);
            setResult("Y", t, ytable[t]);
            setResult("distance", t, dist[t]);
            }
        updateResults;
        saveAs("Results", files[i]+" cone tracking.csv");
        run("Clear Results");
        conechara(xtable,ytable,id,ph,pw);
        return;
}

function expand(x,y,pw,ph) {
            rank = 0;
            for (var n=1; n<10; n++) {
                    makeRectangle(x-((n*10)/2), y-((n*10)/2), n*10, n*10);
                    run("Analyze Particles...", "exclude add");
                    if (roiManager("count")>0) {
                            roiManager("measure");
                            dist = newArray(nResults);
                            for (u = 0; u<nResults; u++) {
                                                                    dist[u]=    sqrt(pow(
                            (x*pw)-getResult("XM", u),2)+pow( (y*ph)-getResult("YM", u),2) );
                            }
                            Array.getStatistics(dist, min);
                            for (i=0; i<lengthOf(dist); i++) {
                    if (dist[i]==min) {
                    rank = i;
                    }
                            }
                            roiManager("deselect");
                            roiManager("delete");
                            run("Select All");
                            run("Clear");
```

```
            }
        }
        return rank;
}
```

Figure S10. Full code of our custom-tailored FIJI macro "Growth cone characteristics".

Movie S1. Rescue of mitochondrial motility in distal axons of ALS FUS mutants through MS at 10 Hz. Shown ist the FUS 495QfsX527 mutant from the ALS pool versus healthy Ctrl1 (Table 1) as representative examples. Corresponds to Fig. 2b., Mitotracker.

Movie S2. Rescue of lysosomal motility in distal axons of ALS FUS mutants through MS at 10 Hz. Shown is the FUS 495QfsX527 mutant from the ALS pool versus healthy Ctrl1 (Table 1) as representative examples, corresponds to Fig. 2b.
